# The impact of emotional labor on emotional exhaustion of social workers: a test of the mediating effect of perceived job demands

**DOI:** 10.3389/fpubh.2026.1823541

**Published:** 2026-06-19

**Authors:** Huan Fu, Yu Tian, Jianzhen Zhao, Lizhen Wei, Haiquan Chen

**Affiliations:** 1 Department of Social Work, Guangdong Baiyun University, Guangzhou, China; 2 School of Business, Sun Yat-sen University, Guangzhou, China; 3 Department of Business Administration, Guangdong Baiyun University, Guangzhou, China; 4 School of Management, Jinan University, Guangzhou, China

**Keywords:** emotional exhaustion, emotional labor, mediate association, perceived job demands, social workers

## Abstract

**Introduction:**

Social work has entered a critical period of high-quality development. As the main force participating in social governance and providing professional services, social workers directly affect the effectiveness of social governance. However, the emotional demands inherent in their work make them vulnerable to emotional exhaustion. Drawing on the theoretical perspective of emotional labor, this study explores the association between social workers’ emotional labor and emotional exhaustion, with particular attention to the mediating role of individuals’ perception of job demand. The central aim is to move beyond a general understanding of emotional labor by deconstructing the antagonistic dynamics among its internal strategies.

**Methods:**

An empirical investigation was conducted with a sample of 399 front-line social workers. The study measured participants’ emotional labor strategies, their perception of job demand, and their levels of emotional exhaustion. Mediation analysis was employed to examine the pathways linking emotional labor to emotional exhaustion through job demand, and moderation analysis was used to assess the role of supportive resources in the work setting.

**Results:**

The findings reveal that social workers generally exhibit a high level of emotional exhaustion, with deep acting being the predominant emotional labor strategy. Job demand plays a significant mediating role between emotional labor and emotional exhaustion, and this mediation displays notable “strategy heterogeneity”: Surface acting is positively associated with emotional exhaustion through increased work demands. Deep acting may predict lower emotional exhaustion by reducing the perception of work demands, thereby generating a “shielding effect” against emotional exhaustion. Furthermore, supportive resources in the work setting moderate the effects of different emotional labor strategies in fundamentally different ways.

**Discussion:**

By deconstructing the antagonistic dynamics of the internal strategies of emotional labor, this study provides a more precise and practically valuable perspective for understanding and intervening in social workers’ job burnout. The identification of “strategy heterogeneity” and the “shielding effect” of deep acting suggests that interventions should be tailored to specific emotional labor strategies rather than treating emotional labor as a unified construct. These insights offer practical guidance for reducing emotional exhaustion among front-line social workers and, in turn, enhancing the effectiveness of social governance.

## 1 Introduction

In recent years, with the increasing complexity and pressure in the social service sector, the emotional labor burden faced by social workers has intensified. This phenomenon is closely linked to the development of occupational burnout (1, 2). Social work is inherently a “labor-intensive” and “emotion-intensive” profession. When dealing with complex client relationships, social workers must invest significant emotional energy. Interacting with others often requires them to regulate and manage their own emotions to meet organizational expectations or client needs. This process not only depletes individuals’ psychological and physiological resources but may also affect their job satisfaction, mental health, and even career development. Against this backdrop, precisely identifying the pathways and boundary conditions through which emotional labor influences emotional exhaustion has become a crucial breakthrough in breaking the vicious cycle of high turnover rates and insufficient service effectiveness.

The practice of social work inherently involves rich emotional dimensions. Emotional labor, linked to autonomous control processes and implicit exploitation, encompasses the ability to regulate and manipulate social interactions, emotions, and thoughts (3) and manage one’s own emotions (4). Its labor process stems from the continuous flow of personal intuition, manifested through external facial expressions, body language, gestures, and even behavioral shaping, ultimately outputting internal feelings and personal cognitions to achieve personal self-valorization. Emotional labor is primarily divided into “surface acting” and “deep acting” (5). Surface acting is often associated with a significant depletion of psychological resources and is regarded as an important predictor of emotional exhaustion. In contrast, deep acting may enhance a sense of professional fulfillment through authentic empathic interactions, thereby mitigating depersonalization tendencies. Within the emotional labor framework, research further distinguishes genuine emotional expression (6). Emotional labor in social work primarily manifests as social workers providing emotional support and expression to clients in practice, in exchange for social recognition and professional identity.

Occupational burnout, as the ultimate manifestation of long-term depletion of emotional resources, is theoretically and operationally characterized by three dimensions: exhaustion, cynicism, and reduced professional efficacy (7). This framework has become central to current research and is widely used for burnout assessment across various professions (8). Among these, emotional exhaustion is considered the core dimension and initial manifestation of burnout, characterized by the excessive depletion of emotional resources, leading to fatigue, numbness, and compassion fatigue (9).

It is within this context that the study focuses on social workers as a specific occupational group, posing the following core questions: Do different emotional labor strategies adopted by social workers correspond to varying degrees of emotional exhaustion? What is the mechanism underlying this effect? Addressing these questions aims to fill the research gap regarding the multi-dimensional and contextualized relationship between emotional labor and emotional exhaustion, while also providing targeted intervention directions for social work practice. This can help social workers in high-emotional-demand environments mitigate burnout by enhancing the resource-generating mechanisms of deep emotional action and genuine emotional expression.

## 2 Research hypotheses

Different emotional labor strategies have different effects on job burnout, especially emotional exhaustion, although some debate exists. A common consensus among scholars is that surface acting leads to cognitive dissonance and continuous depletion of psychological resources because it requires maintaining an internal-external inconsistency between “real feelings and required expressions,” which directly and significantly exacerbates emotional exhaustion (10). However, regarding the effects of deep acting, some studies suggest that deep acting can enhance occupational identity and sense of gain by promoting genuine empathic connection and meaning construction, thereby buffering feelings of burnout (10). Previous studies have further clarified the mechanism of emotion regulation strategies. The surface acting involves behavioral disguise. This continuous cognitive control is associated with severe self-depletion and can predict higher levels of emotional exhaustion (10, 11). Surface acting has a sustained negative impact on employee physical and mental health by mediating emotional inconsistency (12). In contrast, deep acting aligns internal feelings with occupational demands through cognitive restructuring. This psychological consistency not only replenishes psychological resources through positive external feedback but also alleviates the sense of alienation brought about by surface acting (13). In summary, the choice of performance strategy influences the nature of emotional labor. Surface acting, as a “resource–related trait” demand, impairs employee well-being, while deep acting demonstrates “resource–related trait,” significantly enhancing personal sense of achievement and improving service performance (11). While existing research has focused on the relationship between emotional labor strategies and job burnout, it lacks a systematic examination of the differential impact mechanisms of different strategies. It has also failed to systematically reveal how emotional labor strategies indirectly affect emotional exhaustion by influencing individuals’ perceptions of the work situation.

Based on this, this study proposes the following research hypothesis H1: The association patterns between different emotional labor strategies and emotional exhaustion show significant differences. Among them, surface acting is positively associated with emotional exhaustion; deep acting is negatively associated with emotional exhaustion.

Existing research has explored the relationship between emotional labor strategy selection and emotional exhaustion. However, within the context of emotional labor, an individual’s perception of job demand, especially the cognitive evaluation of emotional requirements, refers to the extent to which an individual perceives the need to suppress, fake, or regulate their own emotions at work (14). Job demand refers to aspects of work that require individuals to continuously expend physical, psychological, or emotional effort (Bakker and Demerouti, 2007). The perception of high-intensity work demands is related to the physical and mental energy consumption of individuals. Such consumption may indicate a higher risk of emotional exhaustion (15). Of course, not all job demands produce the same negative consequences; the key lies in an individual’s cognitive evaluation of the nature of the demand. For example, obstacle-related needs, such as role ambiguity, can generate intense emotional depletion because the perceived path to goal achievement is blocked (16). In contrast, while challenge-related needs are also consumptive, if individuals perceive them as contributing to professional growth, the negative impact on burnout may be partially offset by the sense of accomplishment they inspire (17). However, long-term perceptions of high job demand, if lacking corresponding support resources, will eventually lead to job burnout. Moreover, perceived workload is the most direct predictor of emotional exhaustion (1). When individuals perceive high job demand accompanied by low job autonomy, psychological stress reaches its peak, thus significantly accelerating depersonalization (18). An individual’s perception of job demand is a psychological incentive for the emergence of job burnout. Research has found that when employees perceive higher emotional job demand, surface acting has a stronger impact on emotional exhaustion (Grandey et al., 2007). Individual coping strategies and behaviors can influence their perceptions of job demand and job resources, thereby affecting health and motivational outcomes (15).

Therefore, this study proposes a research hypothesis H2, that perceived work requirements may be a mediating variable for predicting emotional exhaustion through emotional labor strategies.

To understand the complete picture of the indirect statistical association consistent with mediation of perceived work requirements, it is also necessary to consider which factors may influence an individual’s perception of job demand, that is, which factors may act as moderating variables, affecting the strength of the mediating path. The configuration balance of work demands and resources is a key framework for understanding job burnout (19). Emotional demand is regarded as a specific job demand. If employees lack sufficient resources, such as autonomy or social support, long-term emotional investment will significantly induce emotional depletion. Personal resources, such as self-efficacy (20), psychological capital (21), and emotional intelligence (22), may moderate the association with emotional labor strategy on perceived work requirements. Individuals with higher emotional intelligence may expend fewer resources when performing emotional labor, resulting in a lower negative perception of job demand (23, 24). Employees with higher emotional intelligence are more effective when using deep acting, which reduces the perception of emotional job demand and the level of emotional exhaustion (25). Organizational resources, such as organizational support and job autonomy, may buffer the association with emotional labor strategy on perceived work requirements (26). When employees perceive higher organizational support, the negative impact of surface acting on perceived work requirements and emotional exhaustion is weakened (27). Job resources can buffer the association with job demand on health impairment (15). Regarding job characteristics, the frequency of emotional labor, intensity, and duration in different work situations may moderate the mediating path (28).

**Figure 1:**
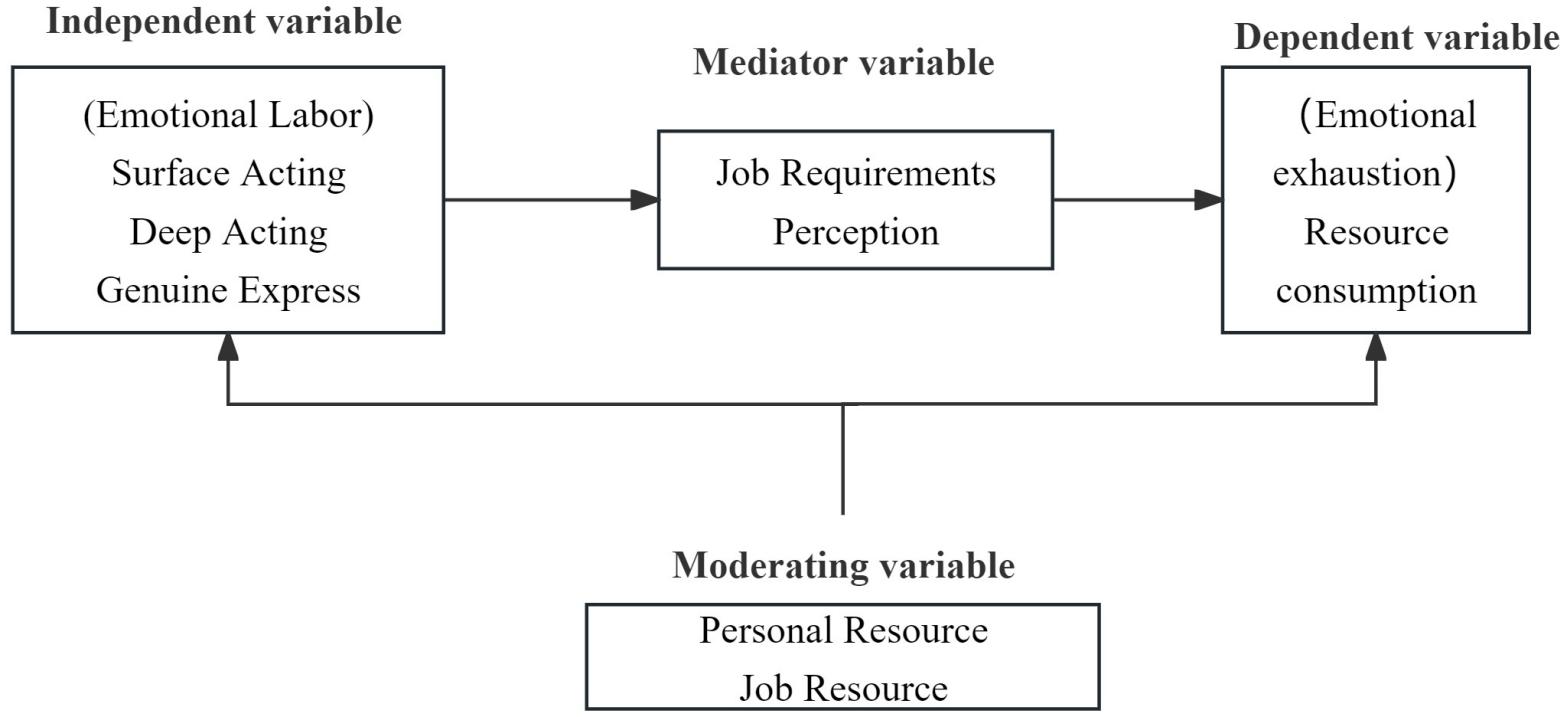
Schematic diagram of the research analysis framework.

In occupations with high emotional demands, the association with emotional labor strategies on perceived work requirements may be more pronounced (29). Accordingly, research hypothesis 3 is proposed: H3: The moderating effect of resource variables on the relationship between emotional labor strategies and perceived work requirements differs depending on the type of strategy (Figure 1).

This analytical framework posits that in the relationship between surface acting and emotional exhaustion, perceived job demands play a crucial mediating role. This study takes emotional labor strategies as the independent variable, emotional exhaustion (a dimension of occupational burnout) as the dependent variable, and perceived job demands as the mediating variable. Specifically, surface acting requires continuous cognitive monitoring; this resource depletion subjectively constructs higher perceptions of workload. In contrast, deep acting reduces the cognitive cost of emotion regulation through value internalization, thereby buffering the subjective perception of job demands. Furthermore, during interactions with clients, clients’ high emotional demands influence the social worker’s individual “demands” (30). Emotional support, organizational resources, and individual emotion management capabilities constitute “resources.” The interaction between demands and resources determines the speed and severity of burnout onset (31).

## 3 Research design

In this study, SPSS 31.0 software and the PROCESS V4.2 program were used for data processing and analysis. Descriptive statistics and Pearson correlation analyses were first conducted to describe the main variables and their bivariate associations. Reliability was assessed using Cronbach’s alpha coefficients, and the Kaiser-Meyer-Olkin test was used to evaluate the suitability of the data for factor analysis.

To examine H1, hierarchical regression analysis was conducted. Respondents’ gender, age, and years of social work experience were entered as control variables, followed by surface acting, deep acting, and genuine emotional expression. Because the study used a cross-sectional observational design, the regression coefficients were interpreted as statistical associations rather than causal effects.

To examine H2, Model 4 of the PROCESS macro was used to test whether perceived job demands accounted for the statistical associations between emotional labor strategies and emotional exhaustion. Bootstrap confidence intervals were estimated using 5,000 resamples. These analyses were interpreted as indirect associations consistent with mediation rather than as evidence of temporal or causal mediation.

To examine H3, Model 7 of the PROCESS macro was used to test whether job resources and personal resources moderated the association between emotional labor strategies and perceived job demands, and whether the indirect association between emotional labor strategies and emotional exhaustion through perceived job demands varied across resource levels.

To reduce the potential influence of common method bias, several procedural remedies were adopted during data collection, including anonymous responses, voluntary participation, and reverse-scored items. In the statistical analysis stage, Harman’s single-factor test was first conducted as an initial diagnostic. Because Harman’s test alone is insufficient to rule out common method bias, supplementary sensitivity analyses were conducted. Specifically, the first unrotated factor score was saved as a common method factor and included as an additional covariate in the PROCESS Model 7 analyses to examine whether the interaction paths remained robust after controlling for this factor. The CMB-adjusted analyses were treated as conservative sensitivity checks rather than replacements for the main models, because the extracted common method factor may capture both method-related variance and substantive psychological variance.

The research measurement tool was a self-compiled integrated questionnaire, comprising four sections. The first section covered sociodemographic and work background information. The second section was an emotional labor strategy measurement scale adapted from Diefendorff’s emotional labor scale (6), covering three dimensions: surface acting, deep acting, and natural expression, with a total of 12 items. The Cronbach’s alpha coefficient for the emotional labor scale was 0.820, indicating high reliability. The third section was a job burnout scale, using the Maslach Burnout Inventory (1). The scale consisted of 22 items, including three dimensions: emotional exhaustion, depersonalization, and low personal sense of achievement. This study adheres to the 7-point rating scale in the original questionnaire. However, in terms of scoring methods, a linear transformation was applied to the original 7-point frequency scale (0 = never, 6 = every day), adjusting it to a 1-to-7 point scale. Specifically, 1 was added to all original scores, meaning “never” is scored as 1 point, “a few times a year or less” as 2 points, and so on, with “every day” being scored as 7 points. This adjustment was made primarily to facilitate data entry and subsequent statistical analysis, and to maintain consistency in form with the commonly used 1-to-7 point Likert scale in other studies. The personal sense of achievement dimension was measured positively and reverse-processed before statistical analysis; higher scores indicated a stronger job burnout feeling. The Cronbach’s alpha coefficient for the overall job burnout scale was 0.862, with a reliability of 0.892 for the emotional exhaustion section, indicating high reliability. The fourth part was the influencing factors scale, including three subsystems: job demands, job resources, and individual resources. The scale was a localized revision based on the JD-R model. The perceived work requirements dimension covered workload, emotional requirements, emotional display rules, and interaction characteristics. The job resources dimension included leadership support, job autonomy, and feedback development. All were scored on a 5-point Likert scale, with an overall reliability of 0.827. The Cronbach’s alpha coefficient for the job demands section was 0.852, indicating strong reliability for this measurement section. The control variables included respondents’ gender, age, and years of social work experience. Other occupational variables, such as employment type, urban or rural service setting, caseload size, and weekly working hours, were not collected in the current survey and therefore could not be included as covariates. This limitation is acknowledged in the Discussion section.

This study targeted frontline practicing social workers as research subjects, and data collection was conducted using a combined sampling method of convenience sampling and snowball sampling. For the recruitment process, between January and February 2026, electronic questionnaires were generated via mainstream online survey platforms. The questionnaire links and invitation letters were first distributed within several social work service agencies, followed by broader dissemination through social workers’ professional networks and WeChat communities using the snowball sampling approach. To control for common method bias and protect participants’ privacy, the questionnaires were completed in full anonymity, and consistent with the original protocol, no personally identifiable information was collected. In terms of procedural settings, the system backend enabled unique restrictions on IP addresses and devices to ensure that each participant could only respond once. Moreover, to ensure the representativeness of the sample and the quality of the data, this study established a strict participant screening procedure. The inclusion criteria were as follows: (1) self-reporting as a currently employed frontline social worker; (2) being at least 18 years of age; (3) having a full understanding of the research purpose, signing an online informed consent form, and voluntarily participating in the survey. During the data cleaning phase, strict quality control processes and exclusion criteria were implemented, with the following types of data specifically excluded: (1) incompletely answered questionnaires; (2) questionnaires with significantly abnormal response times; and (3) questionnaires with obvious patterned responses or clear contradictions in logically conflicting items.

Before data analysis, 410 valid questionnaires were obtained. Invalid questionnaires, such as those with excessively short completion times, logical inconsistencies, and patterned responses, were removed. Finally, 399 valid cases were obtained for subsequent empirical analysis. The valid sample included multiple practical fields, such as community development (23.56%), older adults services (21.3%), child and adolescent services (16.29%), social assistance services (9.52%), and family services (6.02%), these service fields improved the coverage of different social work practice contexts in the sample. However, because the study relied on convenience and snowball sampling, the sample should not be regarded as statistically representative of the broader social work workforce.

Among the 399 social workers surveyed, 119 were male (29.82%), and 280 were female (70.18%) in terms of gender. Regarding age, 122 were 25 years old or younger (30.58%), 195 were between 26 and 35 years old (48.87%), 69 were between 36 and 45 years old (17.29%), and 13 were 46 years old or older (3.26%). Concerning education level, 8 had a high school degree or below (2.01%), 64 had an associate degree (16.04%), 295 had a bachelor’s degree (73.93%), and 29 had a master’s degree or higher (8.02%). The gender distribution of the respondents indicates that the number of female practitioners far exceeds that of male practitioners, which is consistent with the industry characteristics of the social work workforce in China being younger and more female-dominated. However, this may limit the generalization of the research findings to male senior social workers. The majority of social workers hold a bachelor’s degree, suggesting that the profession requires a certain level of professional knowledge and skills. Regarding the age distribution of social workers, they are primarily concentrated under the age of 35, mainly in their mid-youth, and are mostly in the career development stage.

This study is a cross-sectional correlation analysis. The path direction is based on the theoretical framework. The results should not be interpreted as causal effects. Under certain strict assumptions, cross-sectional evidence may provide limited clues for causal inference. This paper does not draw causal conclusions based on this evidence.

## 4 Results

### 4.1 Basic situation of social workers’ emotional labor and emotional exhaustion

This study first conducted descriptive statistics and Pearson correlation analysis on the main variables. According to Table 1, the mean score of emotional exhaustion for social workers is 4.01, with a standard deviation of 1.25, indicating a moderate level. This suggests that emotional exhaustion was relatively common in the sample, although the cross-sectional design does not allow conclusions about how such exhaustion developed over time. Although the mean does not show extreme job burnout, the distribution close to the median often implies that the group is at the critical point of stress imbalance, and any marginal increase in work demands may trigger a widespread sense of exhaustion. Regarding the preference for using emotional labor strategies, the mean for deep acting was 4.16 (SD = 0.92), slightly higher than surface acting (*M* = 4.03, SD = 1.08) and genuine expression (*M* = 4.09, SD = 0.87). This distribution reflects that respondents in practice settings tend to respond to clients’ complex needs through emotional internalization and empathy reconstruction, rather than relying solely on external emotional regulation.

**Table 1:** Descriptive statistics and correlation matrix of variables.

Variable	Mean	SD	Job demands	Surface acting	Deep acting	Genuine expression	Job demands
Job demands	3.41	0.6	1				
Surface acting	4.03	1.08	0.337**	1			
Deep acting	4.16	0.92	0.114*	0.312**	1		
Genuine express.	4.09	0.87	−0.016	0.048	0.596**	1	
Emotional exh.	4.01	1.25	0.610**	0.263**	−0.144**	−0.160**	1

Values are means, standard deviations, and Pearson correlation coefficients. **p < 0.01, *p < 0.05. Job Demands, perceived job demands; Surf. Act, surface acting; Deep Act, deep acting; Genuine Express., genuine emotional expression; Emotional Exh., emotional exhaustion.

Correlation analysis further revealed the intrinsic logic between variables. Surface acting was significantly positively correlated with emotional exhaustion (*r* = 0.259, *p* < 0.01), suggesting that emotional labor, as an alienated form of labor, may exacerbate psychological depletion. Conversely, deep acting was significantly negatively correlated with emotional exhaustion (*r* = −0.171, *p* < 0.01), indicating its potential protective function. The correlation between genuine expression and emotional exhaustion was not significant (*p* > 0.05). Regarding the relationship with the mediating variable, surface acting showed a moderate positive correlation with job demands (*r* = 0.312, *p* < 0.01), while the correlation between deep acting and job demands was weaker but still significant (*r* = 0.142, *p* < 0.05). These results provide an empirical basis for subsequent hypothesis testing, preliminarily supporting the heterogeneity hypothesis regarding the consequences of emotional labor strategies.

### 4.2 Testing the effect of emotional labor on emotional exhaustion

To test H1, hierarchical regression analysis was used to examine whether different emotional labor strategies were differentially associated with emotional exhaustion. As shown in Table 2, Model 1 included the control variables, and Model 2 added surface acting, deep acting, and genuine emotional expression. The inclusion of emotional labor strategies improved the explanatory power of the model.

**Table 2:** Regression analysis of emotional labor on emotional exhaustion.

Variable	Model 1 (control variable)	Model 2 (H1 testing)
Gender (0 = F, 1 = M)	0.254 (1.890)	0.270* (2.148)
Age	−0.406*** (−4.086)	−0.360*** (−3.843)
Years in social work	0.129 (1.394)	0.120 (1.389)
Surface acting		0.602*** (6.958)
Deep acting		−0.527*** (−4.484)
Genuine expression		0.022 (0.250)
*R* ^2^	0.054	0.182
*F*	7.516***	14.513***

The data are unstandardized regression coefficients (*t* values); ***p < 0.001, **p < 0.01, *p < 0.05.

Surface acting was positively associated with emotional exhaustion (*B* = 0.602, *p* < 0.001), suggesting that social workers who reported higher levels of surface acting also tended to report higher levels of emotional exhaustion. Deep acting was negatively associated with emotional exhaustion in the main model (*B* = −0.527, *p* < 0.001), suggesting a potential protective association in the present sample. Genuine emotional expression was not significantly associated with emotional exhaustion (*B* = 0.022, *p* = 0.803). These findings partially support H1 and indicate strategy-specific associations between emotional labor and emotional exhaustion. Because the data are cross-sectional, these results should be interpreted as contemporaneous statistical associations rather than evidence that emotional labor strategies cause emotional exhaustion.

### 4.3 Mediation analysis of job demands

To test H2, this study used Model 4 in Hayes’ PROCESS macro for mediation analysis, with Bootstrap sampling set to 5,000 times. According to Table 3, the results revealed two distinct transmission mechanisms.

**Table 3:** Indirect associations through perceived job demands.

Relational model	Effect (*β*)	SE	*t*	*p*	95% BootCI
Surface acting model					
Surf. act → job demands (*a*)	0.284	0.039	7.272	0	[0.207, 0.361]
Job demands → exh. (*b*)	1.234	0.088	13.957	0	[1.060, 1.408]
Direct effect (*c*′)	0.102	0.073	1.39	0.165	[−0.042, 0.245]
Indirect effect (*ab*)	0.351	0.063			[0.234, 0.478]
Deep acting model					
Deep act → job demands (*a*)	0.116	0.045	2.579	0.01	[0.028, 0.205]
Job demands → exh. (*b*)	1.342	0.08	16.8	0	[1.185, 1.499]
Direct effect (*c*′)	−0.46	0.072	−6.385	0	[−0.602, −0.319]
Indirect effect (*ab*)	0.156	0.068			[0.027, 0.291]

Coefficients are unstandardized; bootstrap samples = 5,000.

#### 4.3.1 Surface acting path

Surface acting significantly positively predicted job demands (*a* = 0.284, *p* < 0.001), and job demands significantly positively predicted emotional exhaustion (*b* = 1.234, *p* < 0.001). The indirect effect was 0.351 (Boot SE = 0.063, 95% CI = [0.234, 0.478]). The confidence interval did not include zero, indirecting association consistent with mediation. Notably, after introducing the mediator, the direct effect of surface acting on emotional exhaustion became non-significant (*c*′ = 0.148, *p* = 0.165), the surface acting model showed a positive indirect association with emotional exhaustion through perceived job demands. Surface acting was positively associated with perceived job demands, and perceived job demands were positively associated with emotional exhaustion. The bootstrap confidence interval for the indirect effect did not include zero, indicating an indirect association consistent with mediation in the present cross-sectional model. Because the direct association between surface acting and emotional exhaustion became nonsignificant after perceived job demands was included, the pattern was consistent with a full indirect association in this sample. However, this should not be interpreted as evidence of temporal or causal mediation. This suggests that the association between surface acting and emotional exhaustion can be largely explained by the perception of job demands.

#### 4.3.2 Deep acting path

Deep acting had a weaker but significant positive association with job demands (*a* = 0.116, *p* = 0.010), while job demands positively predicted emotional exhaustion. The indirect effect was 0.156 (95% CI = [0.027, 0.291]). However, the direct effect of deep acting on emotional exhaustion remained strongly negative and significant (*c*′ = −0.460, *p* < 0.001).

For deep acting, the pattern was more complex. Deep acting showed a weak but significant positive association with perceived job demands, and perceived job demands were positively associated with emotional exhaustion. The indirect association through perceived job demands was therefore positive. At the same time, the direct association between deep acting and emotional exhaustion remained negative. This pattern is consistent with inconsistent mediation or a suppression pattern: the demand-related indirect pathway and the direct association operate in opposite directions. This statistical pattern should not be interpreted as proof that deep acting first creates short-term costs and then produces long-term benefits; such a temporal interpretation requires longitudinal or experience sampling data.

### 4.4 Moderated mediation analysis

To test H3, PROCESS Model 7 was used to examine whether job resources and personal resources moderated the association between emotional labor strategies and perceived job demands, as well as the corresponding indirect association with emotional exhaustion. As shown in Tables 4 and 5, the moderation patterns differed by emotional labor strategy and by resource type.

**Table 4:** Moderated mediation analysis results.

Path relationship and moderator	Effect	SE	*p*	95% CI
A. Surface acting model				
Moderator: job resource (JResour)				
Surf. act × JResour (interaction)	0.104	0.048	0.032	[0.009, 0.199]
Index of mod. mediation	0.128	0.068	—	[−0.008, 0.263]
Moderator: personal res. (PResour)				
Surf. act × PResour (interaction)	0.003	0.06	0.96	[−0.114, 0.120]
Index of mod. mediation	0.004	0.082	—	[−0.157, 0.164]
B. Deep acting model				
Moderator: job resources (JResour)				
Deep act × JResour (interaction)	0.22	0.054	<0.001	[0.114, 0.325]
Index of mod. mediation	0.295	0.08	—	[0.136, 0.450]
Moderator: personal res. (PResour)				
Deep act × PResour (interaction)	0.188	0.051	<0.001	[0.089, 0.288]
Index of mod. mediation	0.253	0.075	—	[0.107, 0.401]

**Table 5:** Conditional indirect associations through perceived job demands at different resource levels.

Independent var. and moderator	Mod. level	Indirect effect	Boot SE	95% LLCI	95% ULCI
A. Surface acting					
1. Mod: job resources	Low (*M* − 1SD)	0.258	0.077	0.112	0.416
	High (*M* + 1SD)	0.451	0.081	0.292	0.614
	Index	0.128	0.068	−0.008	0.263
2. Mod: personal resources	Low (*M* − 1SD)	0.346	0.093	0.168	0.531
	High (*M* + 1SD)	0.351	0.079	0.207	0.516
	Index	0.004	0.082	−0.157	0.164
B. Deep acting					
1. Mod: job resources	Low (*M* − 1SD)	0.064	0.07	−0.075	0.199
	High (*M* + 1SD)	0.506	0.117	0.274	0.736
	Index	0.295	0.08	0.136	0.45
2. Mod: personal resources	Low (*M* − 1SD)	0.08	0.072	−0.067	0.221
	High (*M* + 1SD)	0.438	0.121	0.199	0.667
	Index	0.253	0.075	0.107	0.401

Positive indirect effects indicate that the emotional labor strategy is associated with higher emotional exhaustion through higher perceived job demands. For deep acting, the positive indirect pathway through perceived job demands should be interpreted alongside its negative direct association with emotional exhaustion. Thus, the deep acting results indicate an inconsistent mediation pattern rather than a purely protective indirect pathway. Low and high moderator levels correspond to one standard deviation below and above the mean. Bootstrap samples = 5,000.

#### 4.4.1 Surface acting pathway

For surface acting, job resources significantly moderated the association between surface acting and perceived job demands (*B* = 0.104, *p* = 0.032). The simple-slope pattern indicated that the positive association between surface acting and perceived job demands became stronger as job resources increased. However, the index of moderated mediation was not statistically significant because its confidence interval included zero (Index = 0.128, 95% CI [−0.008, 0.263]). Personal resources did not significantly moderate the surface acting pathway. Thus, the results suggest that the demand-related indirect association of surface acting was relatively stable across personal resource levels and was not clearly buffered by resources.

#### 4.4.2 Resource-contingent pattern in the deep acting pathway

For deep acting, both job resources and personal resources significantly moderated the association between deep acting and perceived job demands. Job resources significantly moderated this path (*B* = 0.220, *p* < 0.001), and the index of moderated mediation was significant (Index = 0.295, 95% CI [0.136, 0.450]). Personal resources showed a similar moderating pattern (*B* = 0.188, *p* < 0.001; Index = 0.253, 95% CI [0.107, 0.401]).

Importantly, these conditional indirect effects should not be interpreted as protective indirect effects. As shown in Table 5, the conditional indirect effects of deep acting through perceived job demands were positive and became significant under high-resource conditions. This indicates that higher levels of job and personal resources strengthened the indirect association between deep acting and emotional exhaustion through perceived job demands. In other words, when resources were high, deep acting was more strongly associated with perceived job demands, which in turn were associated with higher emotional exhaustion.

This pattern is consistent with the suppression pattern reported in the mediation analysis. Deep acting showed a negative direct association with emotional exhaustion, but its indirect association through perceived job demands was positive. Therefore, deep acting should not be described as producing a purely protective pathway through perceived job demands. Rather, the results suggest a dual-pathway pattern: deep acting may be associated with lower emotional exhaustion directly, while also being associated with higher perceived job demands and, through this route, higher emotional exhaustion. Resource variables appear to strengthen this demand-related indirect pathway rather than simply buffer it.

Overall, H3 received partial support. Resource variables moderated selected pathways, but their role was strategy-specific and should not be interpreted as uniformly protective. The moderated mediation results describe conditional statistical associations in a cross-sectional model rather than causal mechanisms.

### 4.5 Common method bias sensitivity analysis

To further address the concern regarding common method bias, supplementary sensitivity analyses were conducted by including the common method factor score as an additional covariate in the moderated mediation models. As shown in Table 6, three of the four interaction paths remained significant after controlling for the common method factor. Specifically, the interactions of surface acting with job resources, deep acting with job resources, and deep acting with personal resources remained significant, whereas the interaction between surface acting and personal resources remained nonsignificant.

**Table 6:** CMB sensitivity analyses of interaction paths in moderated mediation models.

Model	Interaction path	*B*	*p*	95% CI	Index	Boot 95% CI
Surface acting × job resources	Surface acting × job resources → job demands	0.125	0.009	[0.032, 0.218]	0.131	[0.016, 0.248]
Surface acting × personal resources	Surface acting × personal resources → job demands	−0.039	0.476	[−0.146, 0.068]	−0.041	[−0.163, 0.090]
Deep acting × job resources	Deep acting × job resources → job demands	0.174	0.001	[0.076, 0.272]	0.188	[0.082, 0.301]
Deep acting × personal resources	Deep acting × personal resources → job demands	0.192	<0.001	[0.110, 0.275]	0.207	[0.111, 0.311]

All models controlled for gender, age, years in social work, and the common method factor score. Bootstrap samples = 5,000.

**Figure 2:**
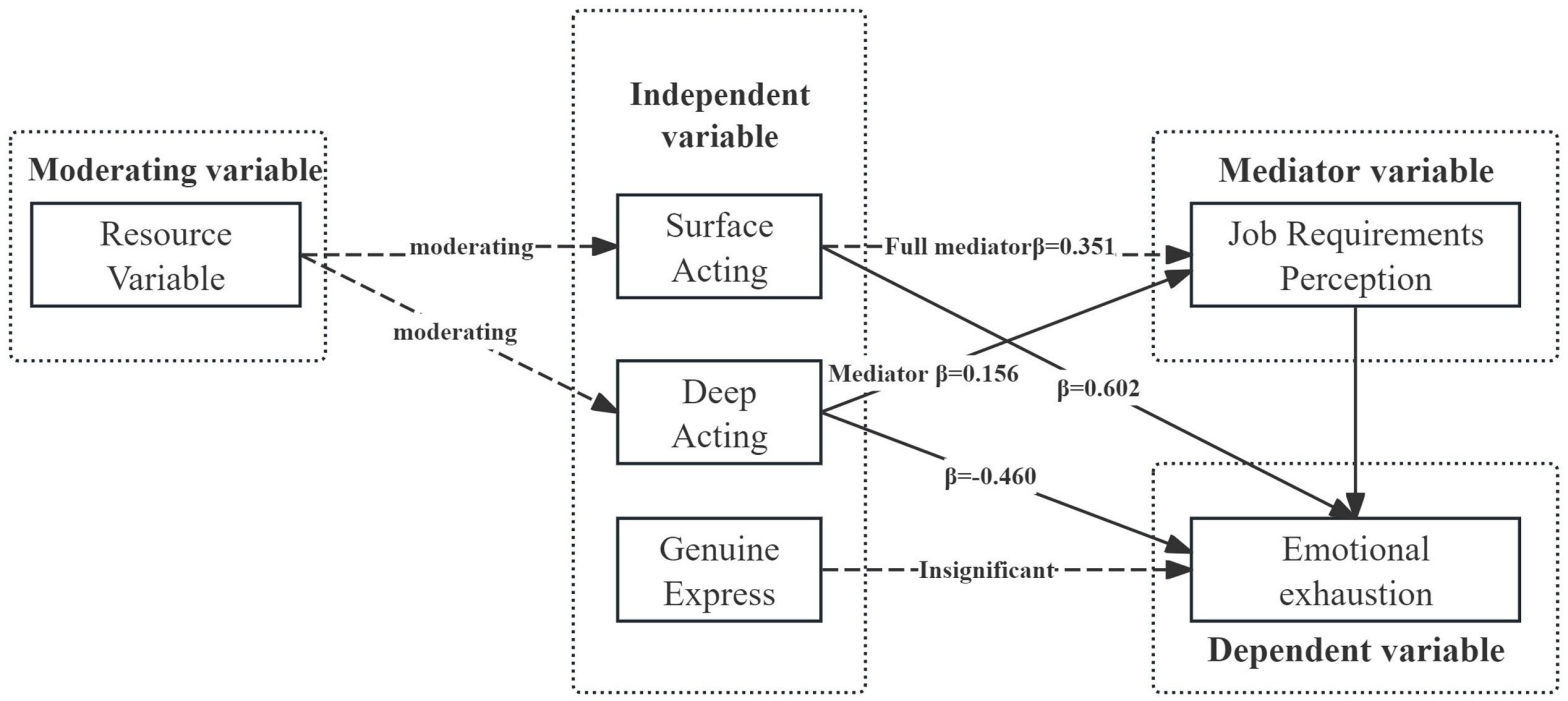
Theoretical model.

The CMB sensitivity analyses produced a pattern broadly consistent with the main moderated mediation models. The resource-contingent pathways involving deep acting remained significant after controlling for the common method factor. However, these results should not be interpreted as replacing the main models. The common method factor extracted from all self-reported items may capture both method-related variance and substantive psychological variance, especially because it was highly correlated with several resource-related variables. Therefore, the CMB-adjusted models should be interpreted as conservative sensitivity checks. They suggest that the key interaction paths were not eliminated after controlling for a common method factor, but they do not fully rule out common method bias (Figure 2).

The study of the positive correlation between surface acting and emotional exhaustion is statistically entirely mediated by perceived job demands. This suggests a possible resource depletion pathway, that surface acting is associated with higher perceived job demands, and the latter is in turn associated with higher emotional exhaustion. The effect of deep acting is more complex: while it also weakly increases risk by raising perceived job demands, its powerful direct protective effect overall overwhelms this indirect risk. This reveals deep acting as a double-edged sword; its internalization process itself requires cognitive effort, potentially increasing burden, but the benefits of successful internalization—emotional authenticity and resource gain—are greater. Ultimately, all these paths converge on the dependent variable, emotional exhaustion. The data show that emotional exhaustion is not caused by emotional labor strategies in a simple linear fashion but is the result of the dynamic interaction between strategy choice, available resources, and cognitive appraisal.

## 5 Discussion

### 5.1 Conditional differentiation mechanism of emotional labor effects and theoretical reconstruction

This study incorporates resources as moderators and perceived job demands as a mediator, proposing a tripartite model of “Strategy Choice—Cognitive Appraisal—Emotional Outcome.” The findings help explain why the relationships between emotional labor strategies and emotional exhaustion vary under different resource conditions. Importantly, the present results suggest that emotional labor strategies are not inherently beneficial or harmful; their effects appear to be contingent upon available resources and cognitive appraisals.

The findings support a contextualized interpretation of emotional labor. Emotional labor strategies should not be treated as inherently beneficial or harmful. Instead, their associations with emotional exhaustion appear to depend on perceived job demands and resource conditions. Surface acting was consistently linked to a demand-related exhaustion pathway. Deep acting, however, showed a more complex pattern: it was negatively associated with emotional exhaustion in the main model, but it also showed a positive indirect association through perceived job demands. This suggests that deep acting may involve both authenticity-related benefits and cognitive or emotional investment costs.

The sensitivity analyses further suggest that deep acting should not be interpreted as an unconditionally protective strategy. After controlling for the common method factor, the direct protective association of deep acting became less stable, whereas the resource-related interaction paths remained significant. This pattern reinforces the view that deep acting may operate through dual mechanisms: it may promote authenticity, professional meaning, and emotional alignment, while also requiring substantial emotional investment and cognitive restructuring under certain conditions. These interpretations should be viewed cautiously given the cross-sectional design.

### 5.2 Differentiation of emotional labor strategies and the suppression effect

The occurrence of emotional exhaustion within social workers’ occupational burnout is closely related to their choice of emotional labor strategies during work. Surface acting is associated with increased risk of emotional exhaustion, while deep acting shows a protective association in this sample. The mediation analysis revealed a suppression effect: the positive indirect effect of deep acting via job demands is outweighed by its negative direct effect. This suggests that, when strategies are not examined separately, the opposing mechanisms may cancel each other out, which may help explain inconsistencies in prior research. It should be noted that these findings are based on cross-sectional data and reflect associations rather than confirmed causal directions. Nevertheless, they underscore the value of distinguishing between different emotional labor strategies in both research and practice.

The findings regarding deep acting should be understood as a statistical suppression pattern rather than a confirmed temporal process. The positive indirect association through perceived job demands may reflect the cognitive and self-regulatory input required by deep acting, whereas the negative direct association may reflect authenticity, professional meaning, or emotional alignment. However, the present cross-sectional design cannot distinguish short-term effort costs from longer-term protective effects, nor can it establish the direction of these associations. Future studies should use experience sampling, daily diary, cross-lagged panel, or intervention designs to examine whether deep acting involves same-day effort costs and longer-term recovery or meaning-building benefits.

### 5.3 Organizational management strategies to reduce situations inducing “surface acting”

This study emphasizes that the focus on reducing social workers’ emotional exhaustion should not be on simply prohibiting surface acting. In fact, in certain high-pressure situations, surface acting is an unavoidable coping strategy for social workers. Simply prohibiting it is not only unrealistic but may increase their psychological burden. The key lies in reducing the situational factors that force social workers to rely on surface acting. Specifically, practical interventions should focus on the following dimensions: reduce the rigidity of emotional display rules: Many social service agencies have strict normative requirements for social workers’ emotional expression. These highly rigid emotional rules force social workers to resort to surface acting when their inner feelings conflict with external requirements. Agency management should re-examine the rationality of emotional display rules, granting social workers greater autonomy in emotional expression and allowing them to express genuine feelings within appropriate boundaries, thereby reducing the passive need for surface acting. Optimize Caseload Allocation and Workload Management: When social workers simultaneously handle a large number of high-difficulty, high-emotional-involvement cases, limited time and energy prevent deep emotional engagement with each client, forcing reliance on surface acting to complete the work. Therefore, reasonably controlling caseload size, scientifically assessing case difficulty, and implementing differentiated allocation are crucial organizational strategies for reducing situations that induce surface acting. Establish an Emotionally Safe Team Culture: In a psychologically unsafe work environment, social workers fear that revealing genuine emotions will be linked with negative evaluations from colleagues or supervisors, making them more inclined toward surface acting. The findings suggest that agencies may consider strengthening supervision, peer support, and psychological resources so that deep acting is less likely to be experienced as an additional demand. However, these practice implications should be interpreted cautiously because the present study is cross-sectional.

### 5.4 Deepening internalization of professional values to foster “deep empathy” strategy generation

Based on the indirect statistical association consistent with the mediation of emotional labor strategies in coping with stress, practical work should focus on enhancing social workers’ emotional transformation capacity. The study found that surface acting triggers cognitive dissonance and accelerates emotional depletion, while deep acting builds a psychological buffer zone. Therefore, the strategy is to guide social workers away from passive defense through masking and toward active adaptation through authentic regulation. Strengthen Value Internalization: Through continuous professional ethics education and value clarification, help social workers truly internalize professional values. When practitioners deeply identify with the principle of “helping others help themselves,” their emotional expression in service will stem more from internal cognitive restructuring rather than merely conforming to external rules. Build Strong Peer Support Networks: Emotional support among colleagues provides a buffer of social support for deep acting, ensuring social workers do not feel isolated when engaging in deep emotional regulation. Agencies can strengthen interpersonal resources for social workers by regularly holding informal exchange activities and forming cross-team learning communities. Enhance Psychological Capital: Provide social workers with systematic psychological capital cultivation programs, including resilience training, mindfulness-based stress reduction courses, and self-care workshops, helping them accumulate internal psychological resources. This ensures they have ample internal resources to draw upon when engaging in deep acting. Develop Supportive Supervision Focused on Emotional Process: The revealed suppression effect indicates that the negative impact of surface acting is often masked by the positive effects of deep acting, making social workers’ psychological distress less detectable. Therefore, supervision should not be limited to administrative functions but should also fulfill emotional support and educational roles. Supervisors should keenly identify signals of cognitive dissonance arising from frequent use of surface acting and provide timely psychological resource replenishment. By incorporating methods like simulations and peer support groups, teach specific techniques for deep acting, helping social workers utilize external resources to assist internal emotion regulation when facing complex cases, thereby enhancing occupational well-being.

The findings of this study collectively point to a core perspective: dealing with emotional exhaustion does not simply involve advocating or prohibiting certain strategies; rather, it may be a systematic task that requires collaborative efforts from multiple aspects. Based on the relevant evidence from this study, effective intervention might focus on updating theoretical models, restructuring organizational ecology, and designing cognitive-level intervention strategies, such as reducing negative contextual factors that are highly associated with superficial role-playing and establishing a resource system that can support the positive role of deep role-playing.

## 6 Limitations and future research directions

Despite the use of theoretically grounded models and supplementary sensitivity analyses, this study has several limitations. First, the cross-sectional and observational design precludes causal inference. All paths in the conceptual model were theory-driven rather than causally identified. Reverse or reciprocal relationships cannot be ruled out. For example, social workers who are already emotionally exhausted may perceive higher job demands or may rely more heavily on surface acting because their psychological resources have been depleted. The temporal limitation is particularly important for interpreting deep acting. The present data capture contemporaneous associations but cannot distinguish short-term cognitive effort from longer-term authenticity-related or meaning-related benefits. Future research should use daily diary designs, experience sampling methods, cross-lagged panel models, or intervention studies to examine temporal ordering and within-person dynamics.

Second, because all variables were measured using self-report questionnaires at a single time point, common method bias cannot be fully ruled out. Although Harman’s single-factor test did not indicate a dominant single factor, this test is not sufficient by itself to exclude common method bias. Common method variance may influence the estimation of interaction paths, potentially inflating, attenuating, or otherwise altering the magnitude of moderated mediation effects. To address this concern, supplementary sensitivity analyses were conducted by controlling for a common method factor score. Three of the four interaction paths remained significant after this adjustment. Nevertheless, these findings should be interpreted as robust statistical associations rather than definitive causal mechanisms. Future studies should use time-lagged, multi-source, or marker-variable designs to better separate substantive effects from method variance.

Third, the sampling strategy limits the generalizability of the findings. The study relied on convenience and snowball sampling within social work networks, which may involve volunteer bias and network homophily. The sample may over-represent younger, more educated, or urban social workers who were more accessible through online professional networks. In addition, some important occupational variables, including employment type, urban or rural service setting, caseload size, weekly working hours, and detailed workload characteristics, were not collected in the current survey. These omissions may have left residual confounding. Future studies should use stratified or multistage sampling, collect more comprehensive occupational and organizational variables, and benchmark the sample against official workforce statistics when available.

## Data Availability

The raw data supporting the conclusions of this article will be made available by the authors, without undue reservation.
